# Interaction between *MLL3* genetic polymorphisms, smoking, and alcohol drinking in laryngeal cancer: a case–control study

**DOI:** 10.1002/cam4.589

**Published:** 2016-01-27

**Authors:** Dong Chen, Liang Gong, Qichuan Jiang, Xuefeng Wang, Bin Zhang

**Affiliations:** ^1^ Department of Otolaryngology Head and Neck SurgeryThe first Affiliated Hospital of Liaoning Medical UniversityNo. 2, Section 5, Renmin Street, Guta DistrictJinzhouLiaoning121001China; ^2^ Department of StomatologyThe First Affiliated Hospital of Liaoning Medical UniversityNo. 2, Section 5, Renmin Street, Guta DistrictJinzhouLiaoning121001China

**Keywords:** Haplotype, laryngeal cancer, *MLL3*, polymorphism

## Abstract

A previous study indicated that *MLL3* genetic polymorphisms were associated with human cancer. However, whether *MLL3* genetic variants are associated with the risk of laryngeal cancer is not clear. This study investigated the association between *MLL3* gene polymorphisms and laryngeal cancer in a Chinese population. Four polymorphisms of the *MLL3* gene (rs6943984, rs4725443, rs3800836, rs6464211) were genotyped using the TaqMan method in 592 patients with larynx cancer and 602 age‐ and sex‐matched noncancer controls. We found that rs6943984 and rs4725443 of the *MLL3* gene were significantly associated with the risk of larynx cancer after Bonferroni correction. The minor allele A for rs6943984 was associated with increased larynx cancer risk (*P* < 0.001, OR = 1.960, 95% CI = 1.587–2.420). C allele frequency (0.151) for rs4725443 was significantly higher in the case group than the control group (0.072, *P* < 0.001). Haplotype analyses showed that haplotypes A‐T‐A‐C and G‐T‐G‐C increased the risk of laryngeal cancer (OR = 2.406, 95% CI: 1.820–3.180, *P* < 0.001; OR = 1.399, 95% CI: 1.180–1.659, respectively), and haplotypes G‐T‐A‐C and G‐T‐G‐T significantly reduced the risk of laryngeal cancer (OR = 0.332, 95% CI: 0.271–0.408, *P* < 0.001; OR = 0.742, 95% CI: 0.607–0.908, respectively). We also found that *MLL3* rs6943984 and rs4725443 polymorphisms had synergistic effects with smoking or alcohol drinking for the risk of laryngeal cancer. This study indicated that *MLL3* genetic polymorphisms and haplotypes were associated with larynx cancer in a Chinese population. There was a mutually synergistic effect between smoking, alcohol drinking, and *MLL3* gene polymorphisms for laryngeal cancer.

## Introduction

Larynx cancer is an important entity of oncology. International data states that larynx cancer accounts for 30–40% of all malignant head and neck tumors 1, 2. The highest incidence of laryngeal cancer occurs between the fifth and seventh decade of life 3, 4. Larynx cancer is a complex disease that results from the interaction between environmental factors and genetics 5, 6, 7, 8. Several environmental factors, including smoking, alcohol consumption, exposure to carcinogens in the work environment, nutrition, and viral infections with human papilloma virus (HPV) and Eostein–Barr virus (EBV), contribute to larynx cancer 9, 10, 11. The progress of molecular biology in the analysis and decoding of DNA proved that a number of genes, called oncogenes, are involved in the mechanisms of carcinogenesis in the larynx. Several DNA repair genes, such as *RAD51*,* XRCC2* 12, *XRCC3* 13, and *RECQL5* gene 14, are associated with larynx cancer.

*MLL3* is a member of the TRX/MLL gene family, and it maps to chromosome 7q36.1. *MLL3* encodes a predicted protein of 4911 amino acids that contain two plant homeodomains (PHD), an ATPase alpha/beta signature, a high mobility group, a suppressor of variegation, enhancer of zeste, trithorax (SET), and two FY (phenylalanine tyrosine)‐rich domains. PHD and SET domain proteins are chromatin regulators, and several of them are altered in cancer 15. Inactivation of MLL3 in mice results in epithelial tumor formation, which suggests that it functions as a tumor‐suppressor gene 16. Additionally, MLL3 is frequently deleted in myeloid leukemias 17, 18, and reports identified somatic mutations in the MLL3 gene in glioblastoma and pancreatic ductal adenocarcinoma 19. However, the relationship between genetic polymorphisms of MLL3 and larynx cancer is not known.

This study established haplotypes of the *MLL3* gene, which consisted of four SNPs (rs6943984, rs4725443, rs3800836, rs6464211) and assessed the relationship between these SNPs and larynx cancer in a Chinese population.

## Material and Methods

### Patients

The Bioethics Committee of the First Affiliated Hospital of Liaoning Medical University approved this study, and each patient gave written consent. All the participants are Chinese Han population and they are not related to each other. Blood samples were obtained from all subjects, including 592 patients with larynx cancer from the Department of Otolaryngology, the First Affiliated Hospital of Liaoning Medical University from May 2006 to June 2014 and 602 cancer‐free age‐ and sex‐matched controls. Patients ranged in age from 44 to 85 years (64.8 ± 8.5), and the control subjects ranged in age from 45 to 83 years (65.0 ± 9.1). Among the larynx cancer patients, there were 315 cases of grade 1, 215 cases of grade 2, and 62 cases of grade 3 in total. Tumor, lymph node and metastasis (TNM) staging indicated that there were 166 cases of stage I, 130 cases of stage II, 201 cases of stage III, 78 cases of stage IVA, and 17 cases of stage IVB.

### Definition of smoking and drinking

As described in pervious study 20, persons reporting regular tobacco use in the previous 6 months were considered as current users. Persons who were ingesting alcohol in the last 6 months were considered to be alcohol users.

### Genotyping

**Figure 1:**
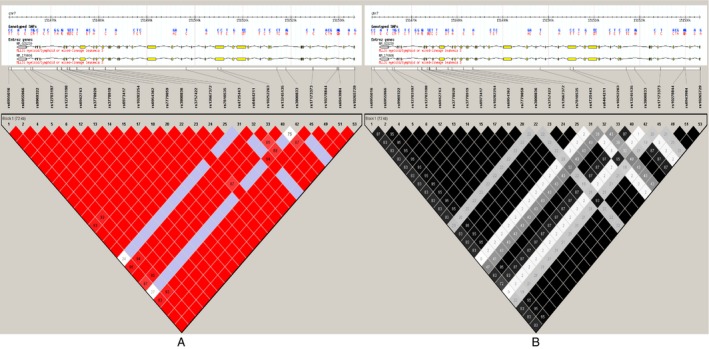
Genetic variations in the human *MLL3* gene. Using Haploview 4.2 software and the HapMap phrase II database, we scanned 53‐genotyped single‐nucleotide polymorphisms (SNPs) in Chinese Han. Linkage disequilibrium (LD) blocks across the locus in Chinese Han. LD block derived using the solid spline method in Haploview 4.2. LD values are shown: (A) |D′| × 100; |D′| color scheme: |D′| = 0: white; 0 < |D′| < 1: shades of pink; |D′| = 1: red; (B) *r* ^2^ × 100; *r* ^2^ color scheme: *r* ^2^ = 0: white; 0 < *r* ^2^ < 1: shades of gray; *r* ^2^ = 1: black.

There are 8070 SNPs for the human MLL3 gene listed in the National Center for Biotechnology Information SNP database (http://www.ncbi.nlm.nih.gov/SNP). We also screened data for the Tag SNPs in the International HapMap Project website (http://www.hapmap.org/). We used Haploview 4.2 software (Harvard University, Cambridge, MA, USA) and the HapMap phase II database obtained four tagging SNPs [rs6943984 (g.152201919G>A), rs4725443 (g.152170176T>C), rs3800836 (g.152149985C>T), rs6464211(g.152176768C>T)] for the Chinese Han subjects using a minor allele frequency (MAF) ≥ 0.05 and linkage disequilibrium patterns with *r* ^2^ ≥ 0.8 as a cutoff. Figure 1 shows that these four SNPs were located in one haplotype block.

Genomic DNA was extracted from peripheral blood leukocytes using a DNA extraction kit (Beijing Bioteke Co. Ltd. Beijing, China). Genotyping was performed using the TaqMan^®^ SNP Genotyping Assay (Applied Biosystems Inc., Foster City, CA, USA) as described previously 20. Two researchers without the knowledge of case or control status blindly conducted all assays. Additionally, approximately 10% of the samples were randomly selected and retested, and the results were 100% concordant.

### Statistical analysis

SPSS 17.0 (Chicago, IL, USA) for Windows was used for statistical analyses. Hardy–Weinberg equilibrium was assessed for each polymorphism using standard chi‐square test or Fisher's exact test. Genotype frequencies in cases and controls were compared using chi‐square tests. Genotype‐specific risks were estimated as odds ratios (ORs) and 95% CI. We performed linkage disequilibrium (LD) analyses and haplotype‐based case–control analyses based on the genotype data of the genetic variations using SHEsis software (http://analysis2.bio-x.cn/myAnalysis.php) 21, 22. Haplotypes with a frequency of <0.03 in the haplotype‐based case–control analyses were excluded. We used a Bonferroni correction to control for the number of variants tested (four) to assess the association of each SNP with laryngeal cancer risk. Therefore, a probability value of 0.0125 was considered to be significant.

## Results

Table 1 shows the participants' characteristics. There were significant differences between the case group and the control group in smoking (*P* < 0.001) and alcohol consumption (*P* < 0.001), especially in men (*P* < 0.001).

**Table 1:** Characteristics of study participants

	Total			Men			Women		
	Case group	Controls	*P*	Case group	Controls	*P*	Case group	Controls	*P*
Number of subjects	592	602		402	411		190	191	
Age	64.8 ± 8.5	65.0 ± 9.1	0.312	65.8 ± 8.8	65.3 ± 9.0	0.543	63.7 ± 8.9	64.0 ± 9.1	0.612
Smoking	431 (72.8)	243 (40.4)	<0.001	341 (84.8)	162 (39.4)	<0.001	90 (47.4)	81 (42.4)	0.006
Alcohol use	302 (51.0)	234 (38.9)	0.087	246 (61.2)	184 (44.8)	<0.001	56 (29.5)	50 (26.3)	0.121
Family history of cancer	69 (11.7)	_		32 (7.9)	_		37 (19.5)	_	

Values are given as mean ± SD or *n* (%).

Table 2 shows the distribution of the genotypes and alleles of the four studied SNPs. The genotype distribution of each SNP was not significantly different from the Hardy–Weinberg equilibrium values (data not shown). Table 2 shows that rs6943984 and rs4725443 of the MLL3 gene were significantly associated with the risk of larynx cancer after Bonferroni correction. The minor allele A for rs6943984 was associated with an increased larynx cancer risk (*P* < 0.001, OR = 1.960, 95% CI = 1.587–2.420). C allele frequency (0.151) for rs4725443 was significantly higher in the case group than the control group (0.072, *P* < 0.001).

**Table 2:** Genotypes and allele distributions in cases and control subjects

SNP	Group	*n*	Genotype			a *P*	Allele		a *P*	OR (95% CI)
rs6943984			AA	AG	GG		A	G		
	Cases	592	40 (0.068)	204 (0.345)	348 (0.588)	<0.001	284 (0.240)	900 (0.760)	<0.001	1.960 (1.587–2.420)
	Control	602	16 (0.027)	135 (0.224)	451 (0.749)		167 (0.139)	1037 (0.861)		
rs4725443			CC	CT	TT		C	T		
	Cases	592	16 (0.027)	147 (0.248)	429 (0.725)	<0.001	179 (0.151)	1005 (0.849)	<0.001	2.287 (1.746–2.995)
	Control	602	6 (0.010)	75 (0.125)	521 (0.865)		87 (0.072)	1117 (0.928)		
rs3800836			AA	AG	GG		A	G		
	Cases	592	111 (0.188)	290 (0.490)	191 (0.323)	0.037	512 (0.432)	672 (0.568)	0.091	0.870 (0.740–1.022)
	Control	602	115 (0.191)	332 (0.551)	155 (0.257)		562 (0.467)	642 (0.533)		
rs6464211			CC	CT	TT		C	T		
	Cases	592	381 (0.644)	194 (0.328)	17 (0.029)	0.032	956 (0.807)	228 (0.193)	0.035	1.235 (1.014–1.505)
	Control	602	363 (0.603)	204 (0.339)	35 (0.058)		930 (0.772)	274 (0.228)		

a *P* value indicated the global difference of genotypes or alleles between the case and control groups. OR values refer to the major allele, such as G of rs6943984, T of rs4725443, G of rs3800836, and C of rs6464211.

**Figure 2:**
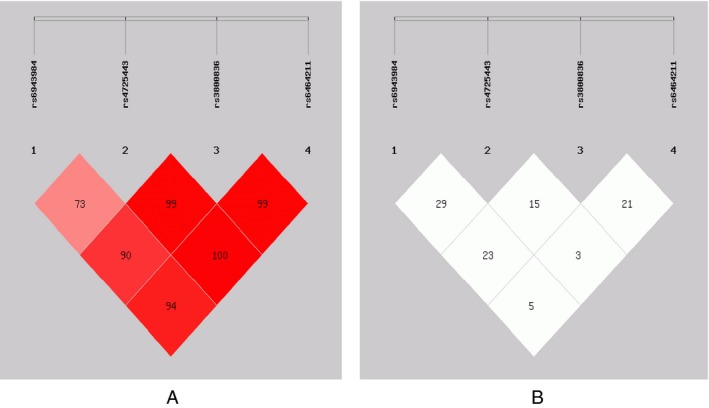
Patterns of linkage disequilibrium in the *MLL3* gene, with their |D′| (A) and *r* ^2^ values (B).

Figure 2 shows the patterns of linkage disequilibrium in the MLL3 gene and the |D′| (A) and *r* ^2^ values (B). All four SNPs are located in one haplotype block because all |D′| are beyond 0.5. All four SNPs were available for the performance of a haplotype‐based case–control study because all of the *r* ^2^ values were below 0.5. The haplotype‐based case–control analyses established haplotypes using four SNPs (Table 3). Haplotype analyses showed that haplotypes A‐T‐A‐C and G‐T‐G‐C increased the risk of laryngeal cancer (OR = 2.406, 95% CI: 1.820–3.180, *P* < 0.001; OR = 1.399, 95% CI: 1.180–1.659, respectively), and haplotypes G‐T‐A‐C and G‐T‐G‐T significantly reduced the risk of laryngeal cancer (OR = 0.332, 95% CI: 0.271–0.408, *P* < 0.001; OR = 0.742, 95% CI: 0.607–0.908, respectively) (Table 3).

**Table 3:** Haplotype distribution of patients and control subjects

Haplotypes	Haplotype frequencies		χ^2^	*P*	OR	95% CI
	Case (*n *= 592)	Control (*n* = 602)				
A C A C	94.71 (0.080)	87.00 (0.072)	0.683	0.408	1.136	0.839–1.538
A T A C	170.28 (0.144)	79.98 (0.066)	39.843	<0.001	2.406	1.820–3.180
G T A C	162.70 (0.137)	395.01 (0.328)	116.79	<0.001	0.332	0.271–0.408
G T G C	443.99 (0.375)	367.99 (0.306)	14.969	<0.001	1.399	1.180–1.659
G T G T	209.00 (0.177)	273.99 (0.228)	8.463	0.004	0.742	0.607–0.908

Global *χ* ^2^ was 229.25, and df = 5 (frequency <0.03 in both control and cases was dropped.) Fisher's *P* value was 2.22 × 10^−15^.

Table 4 describes the influence of the combined effects of rs6943984 and rs4725443 with smoking and drinking on the risk of laryngeal cancer. The GG genotype for rs6943984 in the nonsmoking group (used as a reference, OR = 1) demonstrated that the OR for the risk of laryngeal cancer was 2.327 (95% CI: 1.544–3.507, *P* < 0.001) for carriers of GA + AA. The corresponding OR was 6.679 (95% CI: 4.713–9.466, *P* < 0.001) in the smoking group, which was significantly higher than the OR value obtained by multiplying the risks of laryngeal cancer associated with smoking and the genetic polymorphisms that were obtained in the univariate analyses. Similarly, using the GG genotype and the nondrinking group as a reference (OR = 1), the OR for the risk of laryngeal cancer was 2.175 (95% CI: 1.582–2.989, *P* < 0.001) for carriers of GA + AA in the nondrinking group. The corresponding OR was 5.024 (95% CI: 3.233–7.806, *P* < 0.001) in the drinking group, which was significantly higher than the OR value that was obtained by multiplying the risks of laryngeal cancer associated with drinking and genetic polymorphisms obtained in the univariate analyses.

**Table 4:** Interaction between smoking, drinking, and rs1056827

	GG (TT)				GA + AA (TC + CC)			
	Case group	Control group	OR (95% CI)	*P* value	Case group	Control group	OR (95% CI)	*P* value
rs6943984								
Nonsmoking	101	286	1 (ref.)		60	73	2.327 (1.544–3.507)	<0.001
Smoking	247	165	4.238 (3.806–6.997)	<0.001	184	78	6.679 (4.713–9.466)	<0.001
Nondrinking	144	251	1 (ref.)		146	117	2.175 (1.582–2.989)	0.001
Drinking	204	200	1.777 (1.339–2.359)	<0.001	98	34	5.024 (3.233–7.806)	<0.001
rs4725443								
Nonsmoking	122	306	1 (ref.)		39	53	1.845 (1.160–2.934)	0.009
Smoking	307	215	3.581 (2.726–4.704)	<0.001	124	28	11.107 (7.007–17.607)	<0.001
Nondrinking	155	295	1 (ref.)		135	73	3.519 (2.493–4.968)	<0.001
Drinking	274	226	2.307 (1.775–2.999)	<0.001	28	8	6.661 (2.965–14.965)	<0.001

Using the TT genotype for rs4725443 in the nonsmoking group as a reference (OR = 1), the OR for the risk of laryngeal cancer in the nonsmoking group was 1.845 (95% CI: 1.160–2.934, *P* < 0.001) for carriers of TC + CC. The corresponding OR in the smoking group was 11.107 (95% CI: 7.007–17.607, *P* < 0.001), which was significantly higher than the OR value that was obtained by multiplying the risks of laryngeal cancer associated with smoking and the genetic polymorphisms that were obtained in univariate analyses. Similarly, using the TT genotype in the nondrinking group as a reference (OR = 1), the OR for the risk of laryngeal cancer in the nondrinking group was 3.519 (95% CI: 2.493–4.968, *P* < 0.001) for carriers of TC + CC. The corresponding OR in the drinking group was 6.661 (95% CI: 2.965–14.965, *P* < 0.001), which was significantly higher than the OR value that was obtained by multiplying the risks of laryngeal cancer associated with drinking and the genetic polymorphisms that were obtained in univariate analyses.

These data indicated that rs6943984 and rs4725443 polymorphisms in the *MLL3* gene had synergistic effects with smoking or alcohol consumption for the risk of laryngeal cancer. However, the other two loci did not exhibit synergistic effects with smoking and drinking (data not shown).

Multivariate analysis suggested that after adjustment of confounders such as age, alcohol use, and family history of cancer, the interaction between smoking and rs6943984 and rs4725443 polymorphisms remains significant (*P* < 0.05).

## Discussion

This study found *MLL3* gene polymorphisms and haplotypes that were significantly associated with larynx cancer risk in a Chinese population. To the best of our knowledge, this is the first study to reveal associations of *MLL3* genetic polymorphisms with larynx cancer.

It is generally accepted that larynx cancer is a complex, multifactorial, and polygenic disorder that involves multiple simultaneous environmental and genetic factors. The foundation for human studies examining putative causative genes that may be involved in larynx cancer is based on a candidate gene approach. This approach involves the selection of a functionally relevant gene and a subsequent investigation of its association with the larynx cancer phenotype. A few recent studies revealed that MLL3 genetic polymorphisms were associated with human cancer 23, 24, 25, 26. Our study genotyped four SNPs in the *MLL3* gene in Chinese participants and assessed associations between *MLL3* polymorphisms and larynx cancer using a haplotype‐based case–control analysis. The rs6943984 and rs4725443 distributions differed significantly between larynx cancer patients and control participants. The risk of larynx cancer increased in participants with the A allele of rs6943984 and C allele of rs4725443. We also successfully established haplotypes for the MLL3 gene from the different combinations of the four SNPs. The frequency of the A‐T‐A‐C and G‐T‐G‐C haplotypes was associated with an increased risk for larynx cancer. However, the G‐T‐A‐C and G‐T‐G‐T haplotypes were associated with a decreased risk for larynx cancer.

However, the molecular mechanism related to the association of MLL3 genetic polymorphism and larynx cancer remains unclear. MLL3 is located on chromosome 7q36.1 and has been associated with different types of cancers. MLL3 presents inactivating mutations in medulloblastoma and colorectal cancer 27. Moreover, it shows reduced expression in primary breast tumor samples 24 and radio‐resistant esophageal cancer cell lines 28. A recent study showed that exome sequencing identified a germline mutation in MLL3, producing a truncated protein, in a pedigree of colorectal cancer and acute myeloid leukemia 29. These results suggest that MLL3 acts as a tumor suppressor in cancer development. MLL3 forms a steady‐state complex with ASC‐2, a multifunctional coactivator of nuclear receptors and other transcription factors. This complex called ASCOM interacts with p53 and is required for expression of p53‐target genes in response to DNA damage. These results indicate a role of MLL3 in the DNA damage response pathway through p53 activation 15. The down regulation of MLL3 in larynx cancer may therefore impair the DNA damage response contributing to the proliferation of cancer cell. However, this hypothesis must be further investigated. This study also indicated that subjects who smoke or consume alcohol had a significantly increased risk of laryngeal cancer. These results further confirm that smoking and alcohol consumption are risk factors for laryngeal cancer. Furthermore, our study of the combined effects of gene polymorphisms and the environment on the risk of laryngeal cancer demonstrated that a combination of effects increased the risk of laryngeal cancer, the combination of genetic and environmental factors demonstrated significant synergistic effects of tobacco and alcohol and *MLL3* polymorphisms.

## Conclusion

In conclusion, the present results indicated that larynx cancer was associated with *MLL3* gene polymorphisms. The A‐T‐A‐C and G‐T‐G‐C haplotypes may be useful genetic risk markers, and the G‐T‐A‐C and G‐T‐G‐T haplotypes might be protective factors of larynx cancer in Chinese people. There was a mutually synergistic effect between smoking, alcohol consumption, and *MLL3* gene polymorphisms for laryngeal cancer.

## Conflict of Interest

The authors do not have any conflicts of interest with the content of this manuscript.
